# An Effect of Culture Media on Epithelial Differentiation Markers in Breast Cancer Cell Lines MCF7, MDA-MB-436 and SkBr3

**DOI:** 10.3390/medicina54020011

**Published:** 2018-03-30

**Authors:** Valdis Pirsko, Inese Cakstina, Marta Priedite, Rasma Dortane, Linda Feldmane, Miki Nakazawa-Miklasevica, Zanda Daneberga, Janis Gardovskis, Edvins Miklasevics

**Affiliations:** Institute of Oncology, Riga Stradins University, LV1086 Riga, Latvia; Valdis.Pirsko@rsu.lv (V.P.); marta.priedite@inbox.lv (M.P.); rasmadortane@inbox.lv (R.D.); feldmanelinda2@gmail.com (L.F.); Miki.Nakazawa-Miklasevica@rsu.lv (M.N.-M.); Zanda.Daneberga@rsu.lv (Z.D.); Janis.Gardovskis@rsu.lv (J.G.); Edvins.Miklasevics@rsu.lv (E.M.)

**Keywords:** breast cancer cell line, tumor cells, cultured/substance effects, culture media, gene expression profiling

## Abstract

*Background and objectives:* Cell culture is one of the mainstays in the research of breast cancer biology, although the extent to which this approach allows to preserve the original characteristics of originating tumor and implications of cell culture findings to real life situations have been widely debated in the literature. The aim of this study was to determine the role of three cell culture media on transcriptional expression of breast cancer markers in three breast cancer reference cell lines (MCF7, SkBr3 and MDA-MB-436). *Materials and methods*: Cell lines were conditioned in three studied media (all containing 5% fetal bovine serum (FBS) + hormones/growth factors; different composition of basal media) for four passages. Population growth was characterized by cumulative population doubling levels, average generation time, cell yield and viability at the fourth passage. Transcriptional expression of breast cancer differentiation markers and regulatory transcriptional programs was measured by qPCR. *Results:* Differences in the composition of growth media significantly influenced the growth of studied cell lines and the expression of mammary lineage governing transcriptional programs and luminal/basal markers. Effects of media on transcriptional expression were more pronounced in luminal cell lines (MCF7, SkBr3), than in the basal cell line (MDA-MB-436). Changes in growth media in terms of supplementation and basal medium delayed growth of cells, but improved cell yields. *Conclusions:* The expression of breast cancer cell differentiation phenotypic markers depends on the composition of cell growth medium, therefore cell culture as a tool in phenotypic studies should be used considering this effect. The findings of such studies should always be interpreted with caution. The formulation of cell growth media has greater effect on the expression of phenotypic markers in luminal, rather than basal cell lines. Media containing mitogens and higher vitamin content improved efficacy of cell culture in terms of cell yields, although greatly increased growth times.

## 1. Introduction

Long-lived cancer cell lines, which have been derived from patient tumor cells, have been widely used to explore biology of breast cancer and new therapies in the past and currently remain one of the main models for investigation of breast cancer [1]. Still, there are several recognized gaps in our understanding of cell lines as tools for study of cancer biology, e.g., whether the phenotype of a breast tumor in vivo is maintained in cell culture and how representative cell lines are in reflecting the complexity of the clinical disease [2,3,4].

The complexity of the breast cancer microenvironment with respect to gene expression profiles, signaling pathway activity and drug sensitivity is better recapitulated and modelled by three-dimensional heterotypic culture systems than by adherent cell culture [3]. However, these techniques are time consuming, lack reproducibility and are hard to standardize [5]. Therefore, most molecular profiling studies of breast cancer cell lines have been conducted after culture of cells in medium traditionally used for the breast cancer cell line in the particular laboratory, e.g., in the study of expression profiling of large panel of breast cancer cell lines and in vivo breast tumors by Prat et al. [6] MCF7 and SkBr3 cell lines were cultured in RPMI-1640 with 10% fetal bovine serum (FBS) and the MDA-MB-436 cell line was cultured in DMEM (high glucose) with 10% FBS, while in the study of genomic and transcriptional characterization of breast cancer cell lines by Neve et al. [7], the same cell lines were grown in DMEM medium, McCoy 5A medium and L15 medium, respectively—all media with 10% FBS. The epithelial lineage tracing study performed by Lim et al. [8] propagated mammary and breast cancer cells from human donors in DMEM medium + Ham’s F12 nutrient supplement with 5% fetal calf serum, insulin, glutamine, epidermal growth factor and hydrocortisone. Additionally, high-throughput assays to investigate potential therapeutic agents usually presume conditioning of cell lines to a particular medium, e.g., RPMI-1640 with 5% FBS for discovery of anticancer agents in NCI60 cancer screening panel [9,10]. However, to our knowledge, there are no reports on the extent of differences induced by culture media on the lineage and subtype specific gene expression in breast cancer cell lines. 

Gene expression profiling studies of clinical samples from breast tumors have identified the intrinsic luminal, HER2-enriched, basal, claudin-low and normal breast cancer subtypes [11,12,13], and validity of these subtypes has been shown also in breast cancer cell lines [7,14,15,16,17]. The original method for breast cancer subtype classification on the basis of gene expression profiling [18] has been variously modified and finally simplified to a 50-gene classifier (PAM50) for use in the clinical setting [19]. More recently, mammary epithelial cell lineage tracing and profiling experiments have delineated mammary gland developmental hierarchy and assessed the expression of their specific signatures in tumors of different intrinsic subtypes [8,14,20,21]. These studies have led to the conclusion that breast tumors retain basal or luminal transcriptional programs of the normal mammary epithelium and that these programs are specifically associated with different disease subtypes [22]. Namely, tumors of luminal subtype display mostly the expression signature of normal mature luminal cells [8,11] characterized by high expression levels of luminal lineage regulators *GATA3*, *FOXA1* [23,24], transcription repressor *TBX3* [25], as well as components of the Notch pathway, e.g., *HEY1* and *HES1*, that has been shown to promote luminal differentiation [26,27]. HER2-enriched tumors also express mature luminal cell signature, although the expression of luminal regulators is lower, indicating a less differentiated state [22]. Basal tumors express markers of both luminal and basal mammary lineages [18], while signature analysis revealed their similarity to luminal progenitor cells with enhanced expression of transcription factors *SOX9*, *ELF5*, *FOXM1*, *FOXQ1*, *VGLL1*, *EZH2* [8]. Claudin-low tumors display high levels of expression of mesenchymal markers and regulators of epithelial-to-mesenchymal transition (EMT), and were shown to be best characterized by basal/myoepithelial signature [8] with expression of some regulators of basal lineage, i.e., *ID3*, *TBX2*. Basal/myoepithelial signature has most often been termed mammary stem cell (MaSC) signature, since basal layer of the mammary gland contain cells with properties of MaSC [22]. Although claudin-low tumors are quite rare in clinical practice, they seem to be overrepresented among established breast cancer cell lines [14]. 

In this study, we assessed the effects of basal culture media and hormones/growth factors used in our laboratory to isolate and culture human breast cancer cells from core biopsies of primary tumors. The set of hormones/growth factors used in the study contained mostly mitogens (insulin, epidermal growth factor, estradiol, triiodothyronine) supposedly enhancing proliferation of at least some populations of breast cancer cells, and factors (hydrocortisone, cholera toxin) promoting attachment and spread of the breast cancer cells and formation of focal contacts, i.e., promoting epithelial-like features. The studies the concerning role of these hormones and factors in the culture of breast cancer and/or mammary epithelial cells and their influence on the transcriptional expression of basal or luminal phenotype have been performed mostly with individual hormones/growth factors in isolation and the effects of some hormones have been explored comprehensively, while knowledge concerning the mode of action of other hormones/growth factors (e.g., triiodthyronine) in breast cancer cells with the exception of supposedly proliferation and/or attachment enhancing properties is limited.

The aim of the current study was to investigate how differences in the culture media with respect to basal medium and hormones/growth factors affect transcriptional expression of breast cancer markers and mammary epithelial cell lineage regulatory transcription factors in reference to breast cancer cell lines MCF7, SkBr3, and MDA-MB-436.

## 2. Material and Methods

### 2.1. Breast Cancer Cell Lines

Main clinicopathological features, ER/PR/HER2 status and gene cluster group/transcriptional subtype of the studied breast cancer cell lines MCF7 (ATCC^®^ HTB-22^TM^), SkBr3 (ATCC^®^ HTB-30^TM^) and MDA-MB-436 (ATCC^®^ HTB-130^TM^) are indicated in Table 1.

### 2.2. Basal Growth Media and Supplements

Basal culture media were Dulbecco Modified Eagle’s medium with Ham’s F12 nutrient supplement (1:1) (DMEM:F12 (1:1)), DMEM:F12 (1:3), and RPMI-1640. The basal media DMEM:F12 (1:1) and RPMI-1640 were chosen as all-purpose media traditionally and widely used for propagation of breast cancer cell lines, while DMEM:F12 (1:3) forms the basis for so-called medium F used to promote proliferation of tumor epithelial cells in combination with Rho kinase inhibitor (Y-27632) and fibroblast feeder cells [30]. Nonexhaustive comparison of basal media formulations are shown in Supplementary Table S1. Of the studied basal media, RPMI-1640 was the most rich in terms of energy and carbon sources, but contained the least amounts of amino acids, vitamins and microelements and no lipids or nucleosides. In comparison to DMEM:F12 (1:1), DMEM:F12 (1:3) was enriched in some minerals, vitamins and additional substances. It is worth noting that extracellular levels of calcium can influence the differentiation state of epithelial cells, and studied media in order of decreasing calcium concentration are DMEM:F12 (1:1) > DMEM:F12 (1:3) > RPMI-1640.

The supplements for studied media (hormones and growth factors) and their working concentrations were chosen on the basis of studies performed by Ethier et al. [31,32]: insulin (I; 5 μg/mL), hydrocortisone (HC; 1 μg/mL), epidermal growth factor (EGF; 10 ng/mL), estradiol (E2; 10 nM), transferrin (T; 5 μg/mL), triiodothyronine (T3; 10 μM) and cholera toxin (Ct; 1 ng/mL). The studied media were also supplemented with antioxidant sodium selenite (Se; 50 μM) and buffering agent 4-(2-hydroxyethyl)-1-piperazineethanesulfonic acid (HEPES; 2 nM). 

The final formulations for the control medium (A10) and the studied media (A5, R5, D5) are shown in Table 2. Culture media were coded: A10 (control), A5, D5 and R5. The number in the medium code corresponds to the concentration (%*v*/*v*) of fetal bovine serum (FBS). A10 was the basic cell culture medium consisting of DMEM/F12 (1:1) + 10% FBS with added penicillin/streptomycin. Basis of medium A5 was DMEM:12 (1:1), for medium D5—DMEM/F12 (1:3), and for medium R5—RPMI-1640 (all from ThermoFisher Scientific, Paisley, UK). All media, except A10, were supplemented as described earlier (all supplements from Sigma-Aldrich, Taufkirchen, Germany). For cell line MDA-MB-436 medium A10 was supplemented with 5 μg/mL insulin and 1 ng/mL cholera toxin (Ct).

### 2.3. Cell Culturing and Growth and Viability Analysis

The studied breast cancer cell lines (MCF7, MDA-MB-436, and SkBr3) were conditioned for growth in the three studied culture media for four passages. Each cell line was also propagated in the control medium A10. Cells were cultured in humid chamber at 37 °C, 5% CO_2_. Medium was replaced and cultures were examined in phase-contrast microscope every two days. Total number and viability of cells were determined at each subculture. Cell samples for RNA extraction were obtained after fourth subculture at the studied medium.

Total number and viability of cells were determined by staining with Trypan blue and manual counting in haemocytometer during each subculture. The length of growth of each subculture was documented. 

The effects of media on cellular proliferation rate were evaluated by population doubling level (PDL) at the end of fourth subculture, average generation time during fourth subculture and by cell yield (CY). CY was estimated as CY = T_(cells collected)_/T_(cells seeded)_, where T is the number of cells; PDL was calculated for each subculture (PDL = Log2(CY)). Average generation time during subculture was estimated as average time for all cells in the population to complete one division, i.e., GT = *t*/PDL, where *t* is number of days from seeding to the next subculturing. The level of adaptation of studied cell lines to medium was characterized by the cumulative population doubling levels (PDLs) starting with the first subculture. High levels of adaptation were considered, if the cumulative PDLs at the end of the fourth subculture was at least 15. Data on growth analysis are included in Supplementary Table S2. 

### 2.4. Reverse-Transcription and qReal-Time PCR

Genes for the expression analysis were chosen on the basis of mammary cell tracing experiment by Lim et al. [8], breast cancer cell line profiling experiment by Prat et al. [6], and PAM50 classifier [33] as the ones allowing to detect differentiation related transcriptional programs (regulators) induced or suppressed in breast cancer cells, and as biomarkers, used to distinguish particular subtype of cancer or state of differentiation (luminal markers, basal markers). Studied genes are characterized in Supplementary Table S3.

Total RNA was isolated from one million cells with Qiazol Lysis Reagent (Qiagen, Hilden, Germany) according to manufacturer’s protocol. DNase treatment (ThermoFisher Scientific, Vilnius, Lithuania) for all samples was followed by RNA clean-up with NucleoSpin RNA Clean-up XS columns (Macherey-Nagel, Düren, Germany). Two micrograms of total RNA was used for cDNA synthesis (ThermoFisher Scientific, Vilnius, Lithuania). The quality of cDNA was determined by amplification of *ACTB* and *RNA285S*. Quantitative real-time PCR using 5xHOT FIREPol EvaGreen qPCR SuperMix (Solis Biodyne, Tartu, Estonia) was run on Applied Biosystems Viia7 (Applied Biosystems, Singapore). *ACTB*, *RNA285S* and *GPDH* were used as reference genes. 

### 2.5. Statistical Analysis

Transcriptomic expression analysis was performed in R (version 3.1.2), package “HTqPCR”. The values were normalized using the delta Ct method against three reference genes (*ACTB1*, *RNA28S5*, *GPDH*). To analyze differential expression, the difference to the normalized Ct values in between a couple of media conditions for cell line were subjected to a Student’s *t*-test and evaluated after adjustment of *p*-values by the method of Benjamini and Hochberg with the alpha for false rate discovery of 5%. The genes were considered to be differentially expressed if the *p*-value was ≤0.05. Data (adjusted *p*-value, ddCt and fold change (FC) values) are shown in Supplementary Tables S4 and S5. The FC was calculated using the delta-delta Ct method. 

For analysis, it was assumed that the difference in the expression levels was major, if FC of the expression was less than 0.25 or more than 4.00, moderate, if FC was in the range of 0.25–0.50 or 2.00–4.00, and minor, if FC was in range of 0.50–2.00. We considered only statistically significant (after adjustment of *p*-value by method of Benjamini and Hochberg) and major or moderate differences in the levels of expression.

## 3. Results

### 3.1. Effects of Media on Cell Growth and Morphology

**Figure 1:**
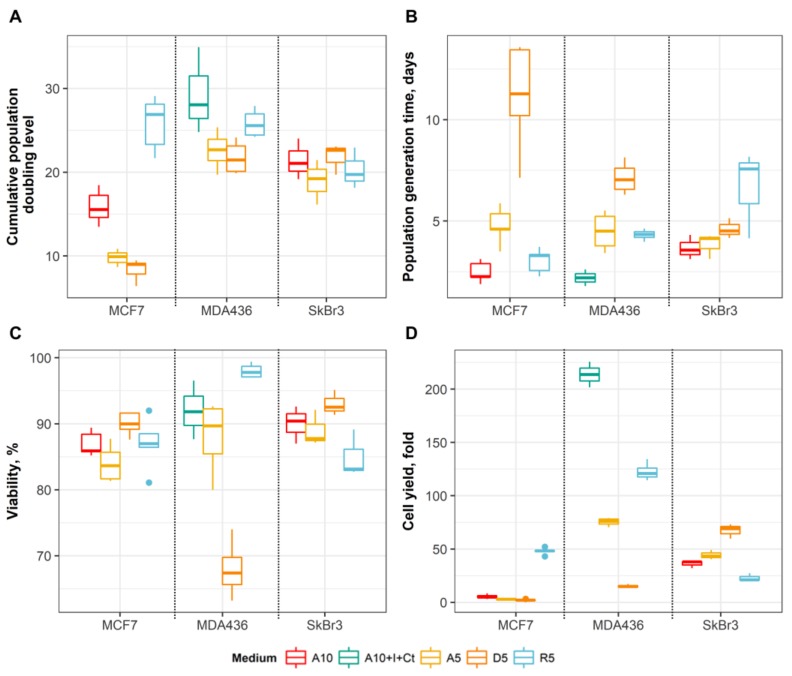
Growth and proliferation of MCF7 (*n* = 5 in each medium), MDA-MB-436 (*n* = 3 in A10 + I + Ct, *n* = 4 in the studied media) and SkBr3 (*n* = 3 in each medium) cell lines in A10, A5, D5 and R5 media during fourth subculture. (**A**) Cumulative population doubling levels in studied media until the end of the fourth passage. (**B**) Cell population generation time. (**C**) The viability of cells after trypsinization of the subculture. (**D**) Cell yields at the end of the subculture.

The characteristics of growth and viability in the studied cell lines during fourth subculture are shown in the Figure 1 and Supplementary Table S3.

Luminal MCF7 cell line achieved high levels of adaptation only in R5 medium (cumulative PDL of 24.87), while the adaptation to A5 and D5 media was low (9.80 and 8.36, respectively) (Figure 1A). A5 and D5 media slowed the growth of MCF7 in comparison to A10 medium (Figure 1B). Moreover, this suppressive effect on proliferation resulted also in lower cell yields (Figure 1D). R5 medium stimulated proliferation of MCF7 cells (generation time of 3.04 days), and substantial increased cell yields (48.02 times). No differences in cell viability were observed for MCF7 cells in the studied media (Figure 1C).

Claudin-low MDA-MB-436 cell line achieved high level of adaptation in all studied media (PDLs of 22.61, 21.74 and 25.82 in A5, D5 and R5 media, respectively) (Figure 1A). All media slowed the growth of MD-MB-436, in comparison to the original A10 + I + Ct medium (Figure 1B). Furthermore, cell yields decreased substantially in all studied media. The suppressive effect on growth of MD-MB-436 in terms of generation time and cell yields was most noticeable in D5 medium, and it was the only medium with low viability of cells (68%) (Figure 1C,D).

HER2-enriched luminal SkBr3 cell line achieved high level of adaption in all the studied media (PDLs of 18.94, 21.80 and 20.27 in A5, D5 and R5 media, respectively) (Figure 1A). All media slowed the growth of SkBr3 cells, in comparison to the control A10 medium (Figure 1B). No differences in cell viability were observed (Figure 1C). Proliferation of SkBr3 cells was stimulated in A5 and D5 media and suppressed in R5 medium in comparison to A10 medium (Figure 1D). Therefore, in contrast to MCF7 and MDA-MB-436 cell lines, A5 and D5 media stimulated growth of SkBr3, while R5 medium suppressed it.

**Figure 2:**
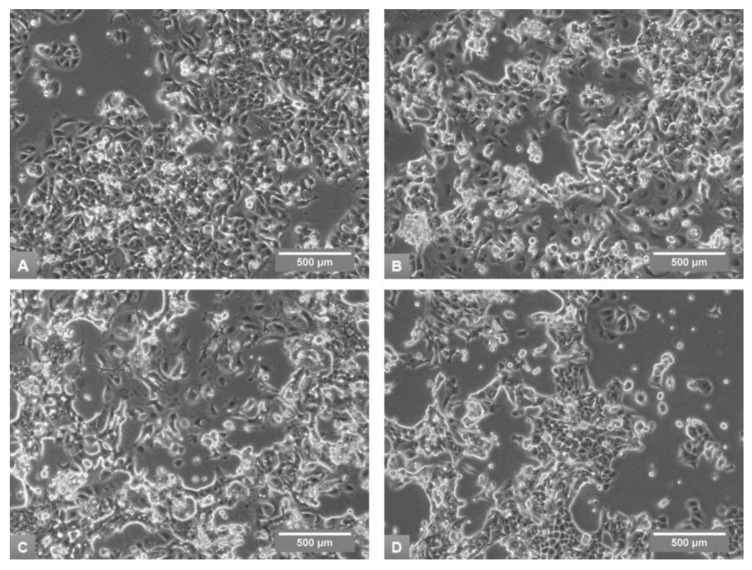
Morphological appearance of MCF7 cells in phase-contrast microscopy on Day 6 of the fourth subculture in A10 (**A**), A5 (**B**), D5 (**C**) and R5 (**D**) media, 100× magnification.

The morphological appearance of MCF7 cells in the studied media also changed prominently (Figure 2). In the original A10 medium MCF7 displayed epithelial morphology with tightly packed, polygonal, flattened cells attached to the substrate, and displaying characteristic “cobblestone” appearance and dome formation at higher densities (Figure 2A).

The growth of MCF7 in A5 and D5 media was characterized by mixture of groups of rounded or irregular cells and fewer, bridge-like clusters with very tightly growing, polygonal cells. The culture showed somewhat detached appearance with increase in free floating or loosely attached cell clusters (Figure 2B,C). Both media also induced increasing formation of mesenchymal-type cells, i.e., single elongated or irregular cells with polarized protrusions. In R5 medium cells retained classical epithelial growth with even seemingly more tight clusters, and the smaller cells in the tight groups gained somewhat rounded polygonal appearance (Figure 2D).

**Figure 3:**
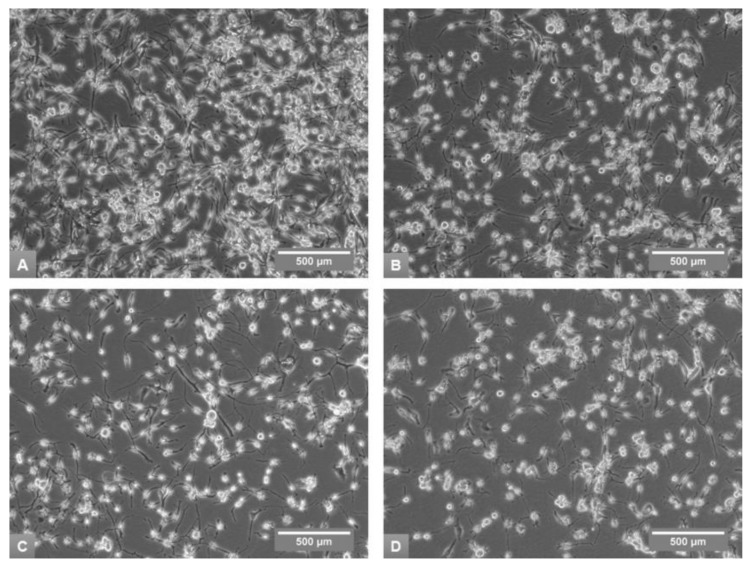
Morphological appearance of MDA-MB-436 cells in phase-contrast microscopy on Day 8 of the fourth subculture in A10 + I + Ct (**A**), A5 (**B**), D5 (**C**) and R5 (**D**) media, 100× magnification.

MDA-MB-436 morphologically is characterized by neuron-type cells, and no noticeable observations were made in addition to the cell density on the same day of subculture (Figure 3).

**Figure 4:**
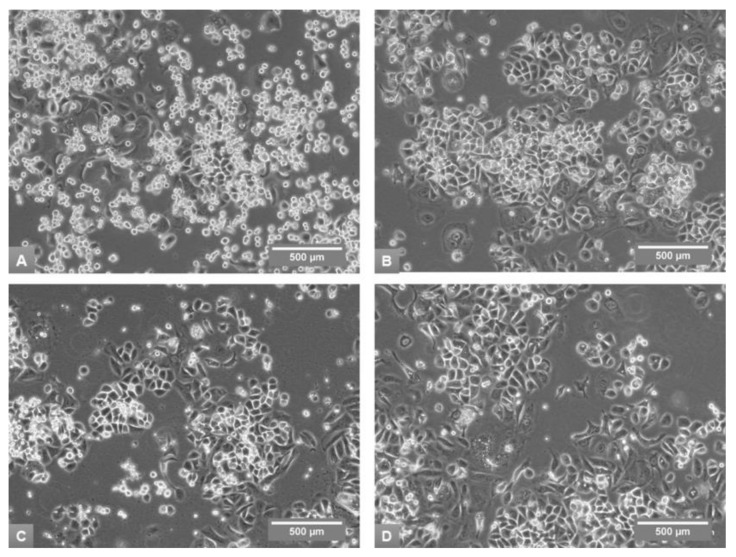
Morphological appearance of SkBr3 cells in phase-contrast microscopy on Day 15 of the fourth subculture in A10 (**A**), A5 (**B**), D5 (**C**) and R5 (**D**) media, 100× magnification.

Morphological appearance of SkBr3 culture in A10 medium was characterized by close groups of polygonal cells, large round multinucleated cells growing mostly in isolation and great amount of free floating or very loosely attached round cells (Figure 4A).

All studied media decreased numbers of free floating and loosely attached cells in comparison to the control A10 medium. Growth in A5 medium displayed increase in the number of large, round multinucleated cells (Figure 4B). The culture in R5 medium appeared somewhat deteriorated, with vacuolization of large cells (Figure 4D). 

### 3.2. Substitution of Serum with Hormones/Growth Factors Shifts MCF7, SkBr3 and MDA-MB-436 Cells Towards Less Differentiated Phenotype

The effects of partial substitution of FBS with hormones/growth factors were evaluated by comparison of the levels of expression of studied genes in A5 medium to the expression of studied genes in A10 medium. Statistically significant major or moderate differences in the levels of expression are shown in Table 3 (values of expression for all genes and *p*-values for comparisons are shown in Supplementary Table S4).

Our results show that the change of growth factor/hormone environment in the studied cell lines had significant impact on the expression of 35 of the 40 studied genes, and that the only genes whose expression was not significantly affected in any of the studied cell lines were basal regulator *ID3*, luminal markers *ESR2* and *NAT1*, and luminal progenitor regulator *FOXQ1*. The pattern of changes in gene expression among cell lines overlapped very little, and the only alteration common to all three cell lines was major or moderate increase in the expression of basal marker keratin 6A gene (*KRT6A*).

Luminal MCF7 cells displayed regulation mostly towards basal phenotype, as evidenced by statistically significant major and moderate increases in the expression of basal regulators (*TP63*, *HEY1*, *IFI16*) and markers (*KRT16*, *KRT16A*, *MIA*, *ERBB2*). The only exception to the trend towards basal phenotype was moderate upregulation of mature luminal regulator *PGR*. 

The response of luminal HER2-enriched SkBr3 cell line was ambiguous with major increase in the expression levels of basal regulators *HEY1* and *IFI16*, and basal markers *ITGB3* and *KRT6A*, and moderate changes in all groups of regulators and markers.

Claudin-low MDA-MB-436 cell line displayed major increase in the levels of basal regulator *TBX2*, and major decrease in levels of basal markers *KRT16* and *KRT17* and luminal marker *MUC1*. MDA-MB-436 also showed moderate increase in the expression of mature luminal regulator *GATA3* and some luminal markers. These changes reflected shift toward basal state of differentiation with increase in some luminal features, probably effected by *GATA3*.

Substitution of A10 medium to A5 medium with growth factor/hormone set employed in this study induced more basal phenotype in all studied cell lines. Addition of growth factors/hormones increased levels of expression of basal regulators *HEY1* and *IFI16* in both luminal cell lines (MCF7, SkBr3), while the most prominent effect in the basal cell line (MDA-MB-436) was major increase in the expression level of basal regulator *TBX2*. One of the main events in MCF7, but not SkBr3 cell line, was major increase in the expression level of the basal regulator *TP63*.

### 3.3. An Effect of Basal Media on Epithelial Differentiation/Breast Cancer Marker Expression Depends on Initial Differentiation State and Genomic Context of the Cancer Cells

The effects of changes in the composition of basal medium were evaluated by comparison of the levels of expression of studied genes in D5 and R5 media to the expression in A5 medium. Statistically significant major or moderate differences in the levels of expression are shown in Table 4 (values of expression for all genes and *p*-values of comparisons are shown in Supplementary Table S5).

As compared to A5 medium, growth of MCF7 in D5 was characterized by minor/moderate shift towards more differentiated state—major decrease in the expression level of *TP63* and moderate downregulation of basal marker *PROCR* and luminal regulator *GATA3*. In comparison to A5 medium, growth of MCF7 in R5 medium showed differentiation shift toward less basal and more luminal state—major decrease in the levels of basal regulators *HEY1*, *SNAI2*, *TP63*, basal markers *ITGB3*, *KRT16*, *KRT6A* and luminal marker *KRT7*, and major increase in the level of luminal marker *SLC39A6*.

The response of the luminal HER2-enriched SkBr3 cell line to D5 medium was somewhat ambiguous with major upregulation of basal regulators *ID3* and *SNAI2*, luminal progenitor regulator *FOXQ1*, mature luminal regulator *HES1* and luminal marker *KRT7* and major decrease in the levels of luminal marker *ESR2*, as compared to A5 medium. Growth of the SkBr3 cell line in R5 medium induced major increase in the levels of mature luminal regulator *GATA3* and major decrease in the levels of basal markers *KRT17* and *KRTAP5-6*. The response of SkBr3 cell line to the various media in terms of expression levels of basal- and luminal/HER2 enriched-phenotype associated genes reflected split in the population of cells with the shift towards both—basal and luminal phenotype.

The growth of claudin-low MDA-MB-436 cells in D5 medium showed little changes in comparison to growth in A5 medium. The only observed changes, as compared to growth in A5 medium, were major and moderate increases in the expression levels of basal markers *KRT6A* and *KRT16*, respectively. Major alterations induced by growth in R5 medium were decrease in the expression levels of luminal progenitor regulator *FOXQ1* and luminal marker *TMEM45B* and increase in levels of expression of basal marker *KRT6A*. Together, major and moderate changes reflected decrease in luminal-like features and shift of MDA-MB-436 cell population towards basal state. 

### 3.4. Transcriptional Profiles

**Figure 5:**
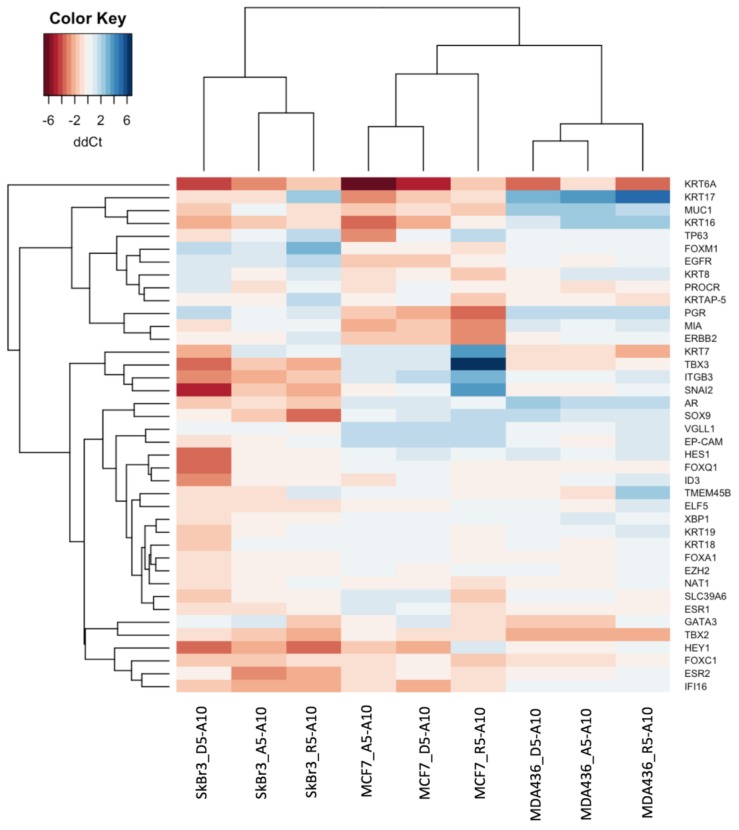
Hierarchical clustering of the differential expression profiles of MCF7, SkBr3 and MDA-MB-436 cell lines across all studied media; here ddCt values are shown in comparison to A10 medium.

Hierarchical clustering of expression alteration profiles induced by substitution of A10 medium to A5, D5 and R5 media clearly show that, despite the observed changes, cell lines retained their identity in terms of mammary lineage regulator and breast cancer marker expression (Figure 5). Interestingly, the changes induced by substitution of basal media and hormones/growth factors put luminal MCF7 and claudin-low MDA-MB-436 cell lines in one cluster, leaving the HER2-enriched SkBr3 cell line as an isolated group. Comparison of Ct values among the same cell line strains grown in different media showed great similarity with correlation coefficient being in range of 0.88–0.97 for the MCF7 cell line, 0.94–0.97 for the SkBr3 cell line, and 0.94–0.98 for the MDA-MB-436 cell line (Supplementary Figure S1). 

Clustering also showed, that phenotypical alterations of the MCF7 cell line in A5 and D5 media were driven mainly by increased expression of *TP63* and related gene *HEY1*, with the most prominent marker change being major increase in *KRT6A* levels. The main drivers for adaptation of the MCF7 cell line to R5 medium were increased expression of mature luminal regulators *ESR1* and *PGR* and decreased expression of mature luminal regulator *TBX3*, reflecting the shift of the MCF7 cell population towards strictly luminal population.

The differences in transcriptional profiles induced by substitution of basal media and hormonal/growth factors in the HER-enriched SkBr3 cell line were numerous and orchestrated by changes in the expression of transcriptional factors of various groups—mature luminal regulators *HES1*, *TBX3*, *GATA3*, luminal progenitor regulators *EZH2*, *FOXQ1*, *SOX9*, and basal regulators *SNAI2*, *HEY1*, *ID3*, *TBX2* with resulting increase in luminal markers *KRT7*, *KRT18*, *MUC1*, *TMEM45B* and *XBP1*, and basal marker *KRT16*. These changes were most distinctively observed in D5 medium. 

The range of changes induced by the studied media in claudin-low MDA-MB-436 cell line was narrower, and was best displayed during growth of the cell line in R5 medium. It was mostly characterized by downregulation of several mature luminal regulators (*ELF5*, *FOXA1*, and *TBX3*), luminal progenitor regulators (*EZH2*, *FOXM1*, and *FOXQ1*) and basal regulator *HEY1*.

## 4. Discussion

The aim of the current study was to investigate how differences in the culture media with respect to basal medium and hormones/growth factors affect transcriptional expression of breast cancer markers and mammary epithelial cell lineage regulatory transcription factors in reference breast cancer cell lines—luminal MCF7, HER2-enriched luminal SkBr3, and claudin-low MDA-MB-436. The results show that differences in growth media in terms of nutrient content and hormones/growth factors have diverse significant effects on phenotype of the cell lines in terms of growth, as shown by growth parameters, and differentiation, as evidenced by transcriptional profiles. Despite separate major or moderate changes in the levels of expression of target genes, any particular cell line grown in various media showed great similarity to itself than any other cell lines, and the correlation coefficients were in the range of 0.88–0.98.

In our study, DMEM:F12 derived media supplemented with hormones/growth factors (A5, D5) suppressed and/or delayed proliferation of luminal MCF7 cell line and claudin-low MDA-MB-436 cell line in terms of average population doubling time and cell yields and enhanced proliferation of HER2-enriched SkBr3 cell line in terms of cell yields, despite shift towards more basal differentiation state as evidenced by changes in transcription profiles. R5 medium, which is composed on the basis of RPMI-1640, showed proliferation enhancing effect on MCF7 cell line with the increase of luminal features in transcription profile, slightly suppressive effect on MDA-MB-436 cell line and was most restrictive to HER2-enriched SkBr3 cell line in terms of proliferation and growth parameters.

The response of SkBr3 and MDA-MB-436 to R5 medium, and also to the substitution of A10 with A5 medium, in terms of gene expression level changes, are more difficult to explain, with up- and downregulation of transcriptional programs (and markers) characteristic for both directions—towards more differentiated and towards less differentiated state. Probably this ambiguous response reflects split of the cell population into marked subpopulations with distinctive properties and phenotypes. The existence of distinct subpopulations of cells within claudin-low and basal-like cell lines has been shown before [6]. There is also a possibility that these results just reflect the complexity in the regulation of epithelial phenotype with several transcriptional programs acting concurrently and the final fate of the population being determined by balance in the interplay of basal, luminal progenitor and mature luminal regulators. To provide comprehensive explanation of the observations presented by this study there is need for further exploration of the translational expression of the studied markers on cell population level that would allow to reveal various subpopulations of cells in the studied cultures. Also, to uncover signaling pathways effecting the observed changes in the phenotype of cultures there is need to expand range of analyzed genes, including epithelial-to-mesenchymal transition markers, tumor-initiating cell markers, and proliferation markers in the analysis panel. If the cell yields are main concern in the culturing, MDA-MB-436 can be cultured in A10 + I + Ct media, and SkBr3—in D5 medium. The highest MCF7 cell yields were obtained from culturing in R5 medium. Nevertheless, in case of MCF7 the phenotypic and transcriptome changes in R5 media should be taken in account in the process of designing and performing various experiments and assays, where phenotype is of great importance. 

Generally, the response to the substitution of DMEM:F12 1:1 (A) medium with DMEM:F12 1:3 (D) or RPMI-1640 (R) media probably depends on the initial differentiation state and genomic context of the studied cell lines with luminal MCF7 cell line showing shift towards more luminal phenotype, luminal SkBr3 cell line showing somewhat uncertain alteration with up- and downregulation of both, basal- and luminal-phenotype associated regulators and markers, and claudin-low MDA-MB-436 cell line displaying shift towards mesenchymal phenotype.

Luminal MCF7 cell line showed most homogeneous response towards change of growth media, therefore it is a good example for significant changes in phenotype induced by changes in growth conditions. 

MCF7 displayed most pronounced shift towards less differentiated state in A5 and D5 media. It was showed not only by transcriptional profile with up-regulation of basal regulators (especially *TP63*) and markers, and down-regulation of mature luminal and luminal progenitor regulators and luminal markers, including moderate decrease in the expression of lineage defining marker *EPCAM* and increase in tumor-initiating cell marker *PROCR*, but also by induction of epithelial-to-mesenchymal like transition observed as increased number of mesenchymal-type cells in A5 and D5 media, as described previously [34]. Our study also allowed to identify probable players in the adaptation of MCF7 cell line to the studied media, i.e., *TP63*, *HEY1*, *TBX3* and *PGR*. Although it has been observed, that high expression of p63 protein is characteristic to breast cancer cells with basal-like features [35], in this study MCF7 cell line was characterized by the highest expression of *TP63* as compared to the other studied cell lines. Increased levels of p63 have been shown to be related to the activation of Notch pathway, involved in the maintenance of stem cell population in various tissues and in the regulation of the differentiation of cells. Some of the Notch signaling pathway downstream targets are Hairy/Enhancer of Split related genes (e.g., *HEY1*) and *Notch1* itself [36]. It has also been shown, that Notch-expressing breast cancer cells display increased cancer stem cell features and can initiate tumor formation in mice [37]. Therefore, activation of Notch pathway could be the case in substitution of A10 medium with A5 and D5 media for MCF7 cells. 

Interestingly, in MCF7 cell line partial substitution of serum with hormones/growth factors resulted also in increased expression of *PGR*, and decreased expression of *ESR1*. It is known, that progesterone receptor modulates the behavior of estrogen receptor α (ERα) [38]. Additional expression of *PGR* in ERα-positive breast cancer cells has suppressed estrogen-mediated proliferation and transcriptional activity of ERα [39]. Therefore, increased expression of *PGR* and decreased expression of *ESR1* in MCF7 conditioned to A5 and D5 media could at least partially explain suppression of growth of the cell line in A5 and D5 media. 

Overexpression of mature luminal regulator *TBX3* in breast cancer cell lines has been observed previously, especially in cells with estrogen-receptor positive status, e.g., MCF7 [40], and it has been shown that estrogen induces *TBX3* expression, increasing number of stem-like cells [41]. Therefore, it can be suggested that growth of MCF7 in R5 medium is characterized by decrease in number of cells with tumor initiating capability, however further studies are needed to confirm this hypothesis. Changes of *TBX3* expression levels in A5, D5 and R5 medium, as compared to A10 medium, is a good example, how nutrients in basal medium influence the expression of cell differentiation governing transcriptional programs.

The impact of individual nutrients, hormones and factors on the transcriptional expression of various markers in culture of breast cancer and/or mammary cells has been studied before, e.g., reports on the effect of estrogen on transcriptional activity in various cell lines [42,43,44,45,46,47], but none of these studies have explored the long-term effects of supplementation with complex set of hormones/growth factors on transcriptomic profile of phenotype defining genes in breast cancer cell lines for exposure longer than five days [45]. Therefore, this is the first characterization of the long-term effects of complex hormone/growth factor set on the expression of breast cancer/mammary markers in breast cancer cell lines. Also, to our knowledge, no studies on the effect of basal media on the expression of the markers have been performed before. 

This study clearly shows that growth conditions employed in various laboratories for propagation of cancer cell lines can have significant major impact on the expression of genes defining the phenotype of the breast cancer cells. Therefore, the possible interference of growth media has to be taken in account in the process of designing and performing various experiments and assays, where phenotype of studied breast cancer cell types is of great importance. 

## 5. Conclusions

The expression of breast cancer cell differentiation phenotypic markers in vitro depends on the composition of cell growth medium, therefore cell culture as a tool in phenotypic studies should be used taking into account this effect and the findings of such studies should always be interpreted in caution. Despite changes in the marker expression levels in the framework of our study cell lines retained their phenotypic identity. The formulation of cell growth media has greater impact on the expression of phenotypic markers in luminal, than basal cell lines. Replacement of fetal bovine serum with hormones/growth factors and substitution of basal medium has significant effect on cellular proliferation rates and cell yields. Media containing mitogens and higher vitamin content improved efficacy of cell culture in terms of cell yields, although greatly increased growth times.

## Figures and Tables

**Table 1:** Characteristics of the studied breast cancer cell lines.

Cell Line	Source *	Tumor Type *	ER *	PR *	HER2 *	Cluster/Subtype **	Complete Growth Medium According to ATCC *
MCF7	PE	IDC	+	+	0	luminal	Eagle’s Minimum Essential Medium + 10% FBS + 0.01 mg/mL hr insulin
SkBr3	PE	AC	0	0	+	luminal/HER2+	McCoy’s 5a +10% FBS
MDA-MB-436	PE	IDC	0	0	0	basal B/claudin-low	Leibovitz’s L-15 + 10% FBS + 10 μg/mL insulin + 16 μg/mL glutathione

PE—pleural effusion; IDC—invasive ductal carcinoma; AC—adenocarcinoma; +—positivity for ER, PR expression or HER2 overexpression; 0—negativity for ER, PR expression or HER2 overexpression; FBS—fetal bovine serum hr – human recombinant; * Information from ATCC (https://www.lgcstandards-atcc.org/) and references therein. ** Information from the following studies: [7,16,28,29].

**Table 2:** Formulations of the control and studied media.

Medium	Basis	FBS (%)	Supplements *
A10	DMEM/F12 (1:1)	10	P/S
A5	DMEM/F12 (1:1)	5	P/S, I, HC, EGF, E2, HEPES, T, T3, Se, Ct
D5	DMEM/F12 (1:3)	5	P/S, I, HC, EGF, E2, HEPES, T, T3, Se, Ct
R5	RPMI1640	5	P/S, I, HC, EGF, E2, T, T3, Se, Ct

* P/S—1% Pen/Strep; I—insulin; HC—hydrocortisone; EGF—epidermal growth factor; E2—17β-estradiol; HEPES—(4-(2-hydroxyethyl)-1-piperazineethanesulfonic acid); T—transferrin; T3—3,3′,5-triiodo-l-thyronine; Se—sodium selenite; Ct—cholera toxin.

**Table 3:** Differential transcription of studied genes in A5 medium, as compared to A10 medium, in all three cell lines (expression level and *p*-values are reported in Supplementary Table S4) *.

Gene Class **	Gene	MCF7	SkBr3	MDA-MB-436
MLR	AR	↓↓	↑↑	↓↓
	ELF5	NS	↑↑	NS
	ESR1	↓	NS	NS
	FOXA1	↓	NS	↑
	GATA3	NS	↓↓	↑↑
	HES1	NS	↑	NS
	PGR	↑↑	NS	NS
	TBX3	↓↓	NS	↑
LPR	EZH2	↓	NS	NS
	FOXM1	NS	↓↓	NS
	FOXQ1	NS	NS	NS
	SOX9	NS	↑↑	↓↓
	VGLL1	↓↓	↓	NS
BR	HEY1	↑↑	↑↑↑	NS
	ID3	NS	NS	NS
	IFI16	↑↑	↑↑↑	↓
	SNAI2	↑	NS	NS
	TBX2	NS	↑↑	↑↑↑
	TP63	↑↑↑	NS	NS
LM	EPCAM	↓↓	NS	NS
	ESR2	NS	NS	NS
	KRT18	NS	↓	NS
	KRT19	NS	NS	↓
	KRT7	NS	↓	↑↑
	KRT8	NS	NS	↓
	MUC1	NS	NS	↓↓↓
	NAT1	NS	NS	NS
	SLC39A6	↓	NS	↓
	TMEM45B	NS	↑	↑↑
	XBP1	NS	NS	↓
BM	EGFR	NS	↓	↑
	ERBB2	↑↑	NS	NS
	FOXC1	NS	↑↑	↑↑
	ITGB3	NS	↑↑↑	NS
	KRT16	↑↑↑	↑↑	↓↓↓
	KRT17	NS	↑	↓↓↓
	KRT6A	↑↑↑	↑↑↑	↑↑
	KRTAP5-6	NS	↑	NS
	MIA	↑↑↑	NS	NS
	PROCR	↑	NS	↑

* Only statistically significant differences shown; ↑↑↑—major up-regulation (fold-change [FC] > 4.0), ↑↑—moderate up-regulation (FC = 2.00–4.00), ↑—minor up-regulation (FC = 1.00–2.00), ↓↓↓—major down-regulation (FC < 0.25), ↓↓—moderate down-regulation (FC = 0.25–0.50), ↓—minor down-regulation (FC = 0.5–1.00)); NS—not significant. ** Gene classes: MLR—mature luminal regulator; LPR—luminal progenitor regulator; BR—basal regulator; LM—luminal marker; BM—basal marker.

**Table 4:** Differential transcription of studied genes induced by basal medium in all three cell lines—comparison of D5 and R5 media to A5 medium (expression level and *p*-values are reported in Supplementary Table S5) *.

Gene Class **	Gene	MCF7		SkBr3		MDA-MB-436	
		D5	R5	D5	R5	D5	R5
MLR	AR	NS	↑	NS	↑	NS	NS
	ELF5	NS	NS	NS	↑	NS	↓↓
	ESR1	↑	↑↑	NS	↓	NS	NS
	FOXA1	NS	↑	↑	↑	NS	↓
	GATA3	↓↓	↑	NS	↑↑↑	NS	NS
	HES1	↓	NS	↑↑↑	NS	NS	↓
	PGR	↑	↑↑	NS	NS	NS	NS
	TBX3	NS	NS	NS	↑	NS	↓
LPR	EZH2	↑	NS	↑	↑	NS	↓
	FOXM1	NS	NS	NS	↓↓	NS	↓
	FOXQ1	NS	NS	↑↑↑	NS	NS	↓↓↓
	SOX9	NS	↓↓	↓↓	↑↑	NS	NS
	VGLL1	NS	NS	NS	↑	NS	NS
BR	HEY1	NS	↓↓↓	↑↑	↑↑	NS	↓
	ID3	NS	NS	↑↑↑	NS	NS	NS
	IFI16	NS	NS	↓	NS	NS	NS
	SNAI2	↓	↓↓↓	↑↑↑	NS	NS	NS
	TBX2	NS	NS	NS	↑↑	NS	NS
	TP63	↓↓↓	↓↓↓	NS	NS	NS	NS
LM	EPCAM	NS	↑	NS	↓	NS	↓↓
	ESR2	NS	NS	↓↓↓	NS	NS	NS
	KRT18	NS	↑↑	↑↑	NS	NS	↓
	KRT19	NS	↑↑	NS	NS	NS	↓
	KRT7	NS	↓↓↓	↑↑↑	NS	NS	↑↑
	KRT8	NS	NS	NS	NS	NS	↓
	MUC1	NS	NS	↑↑	NS	NS	↑
	NAT1	NS	↑	NS	NS	NS	↓
	SLC39A6	NS	↑↑↑	NS	NS	NS	↓
	TMEM45B	NS	NS	↑	↓↓	NS	↓↓↓
	XBP1	NS	↑	↑	NS	NS	NS
BM	EGFR	NS	↓↓	↓	↓	NS	↓
	ERBB2	NS	NS	↑	↓↓	NS	NS
	FOXC1	NS	NS	NS	↓	NS	↓
	ITGB3	↓	↓↓↓	NS	NS	NS	NS
	KRT16	NS	↓↓↓	↑	↓↓	↑↑	NS
	KRT17	NS	NS	NS	↓↓↓	NS	↓
	KRT6A	NS	↓↓↓	↑↑	↓↓	↑↑↑	↑↑↑
	KRTAP5-6	NS	NS	NS	↓↓↓	NS	NS
	MIA	↓	NS	NS	NS	NS	↓
	PROCR	↓↓	NS	NS	NS	NS	↓

* Only statistically significant differences shown; ↑↑↑—major up-regulation (fold-change [FC] > 4.0), ↑↑—moderate up-regulation (FC = 2.00–4.00), ↑—minor up-regulation (FC = 1.00–2.00), ↓↓↓—major down-regulation (FC < 0.25), ↓↓—moderate down-regulation (FC = 0.25–0.50), ↓—minor down-regulation (FC = 0.5–1.00); NS—not significant. ** Gene classes: MLR—mature luminal regulator; LPR—luminal progenitor regulator; BR—basal regulator; LM—luminal marker; BM—basal marker.

## Supplementary Materials

The following are available online at http://www.mdpi.com/1010-660X/54/2/11/s1, Supplementary Figure S1: Correlation plot of Ct values for the expression of studied genes in reference breast cancer cell lines: (A) MCF7, (B) SkBr3, and (C) MDA-MB-436., Table S1: Formulation of basal media, Table S2: Outcomes of MCF7, MDA-MB-436 and SkBr3 cell growth in A10, A5, D5 and R5 media during the fourth subculture, Table S3: Gene information, Table S4: Gene expression delta Cts and fold changes compared to the basal medium A10, Table S5: Gene expression delta Cts and fold changes—comparison of D5 and R5 media to A5 medium.
