# Supplementary material for: An Effect of Culture Media on Epithelial Differentiation Markers in Breast Cancer Cell Lines MCF7, MDA-MB-436 and SkBr3
Source: Medicina (Kaunas). 2018 Mar 30;54(2):11. doi: 10.3390/medicina54020011 (PMC6037242; doi:10.3390/medicina54020011)

Pirsko V, Cakstina I, Priedite M, Dortane R, Feldmane L, Nakazawa-Miklasevica M, Daneberga Z, Gardovskis J, Miklasevics E. "An effect of culture media on epithelial differentiation markers in breast cancer cell lines MCF7, MDA-MB-436 and SkBr3"

Supplementary Figure 1. Correlation plot of Ct values for the expression of studied genes in reference breast cancer cell lines: (A) MCF7, (B) SkBr3, and (C) MDA-MB-436. The upper triangle contains Ct values plotted pairwise against each other for comparison of media. The lower triangles show the correlation between samples.

(A)

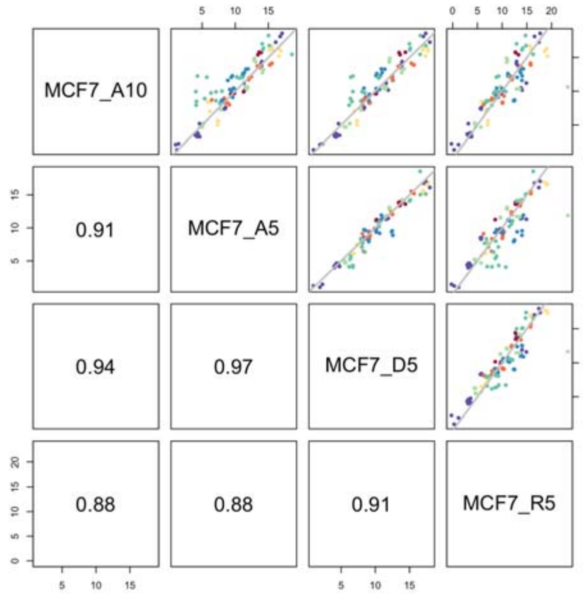

(B)

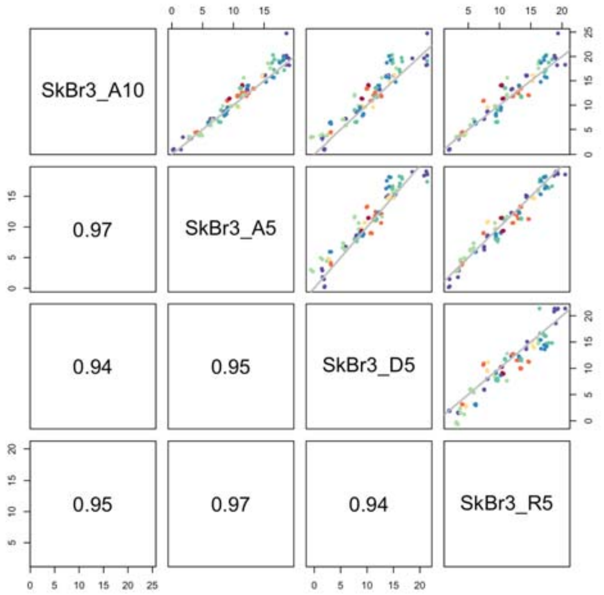

(C)

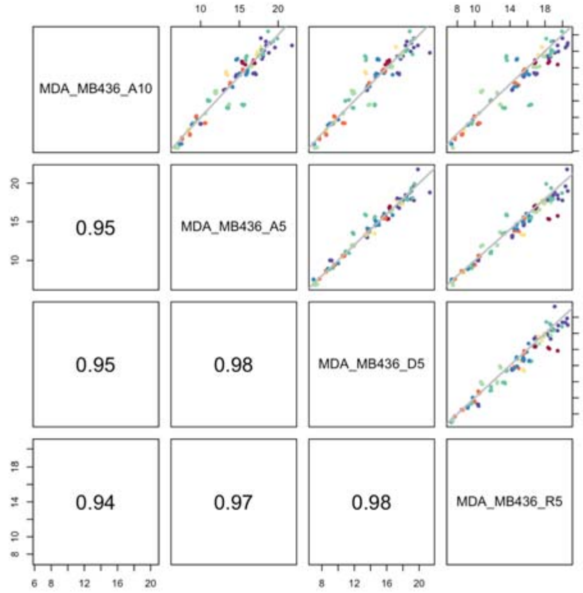

Supplement: Supplementary file 1 [file medicina-54-00011-s001.zip › Supplementary files_after final proofreading/Pirsko et al_SupplFigure01_Correlations.pdf]
