# Supplementary material for: An Effect of Culture Media on Epithelial Differentiation Markers in Breast Cancer Cell Lines MCF7, MDA-MB-436 and SkBr3
Source: Medicina (Kaunas). 2018 Mar 30;54(2):11. doi: 10.3390/medicina54020011 (PMC6037242; doi:10.3390/medicina54020011)
Supplement: Supplementary file 1 [file medicina-54-00011-s001.zip › Supplementary files_after final proofreading/Pirsko et al_SupplTable02_Growth outcomes.pdf]

Pirsko V, Cakstina I, Priedite M, Dortane R, Feldmane L, Nakazawa-Miklasevica M, Daneberga Z, Gardovskis J, Miklasevics E. "An effect of culture media on epithelial differentiation markers in breast cancer cell lines MCF7, MDA-MB-436 and SkBr3"

Supplementary Table 2. Outcomes of MCF7, MDA-MB-436 and SkBr3 cell growth in A10, A5, D5 and R5 media during the fourth subculture

Cell line	Medium	Cumulative population doubling level	Generation time, days	Viability, %	Cell yield, times
**MCF7**	A10	15.86	2.48	87	5.47
	A5	9.80	4.78	84	2.92
	D5	8.36	11.13	90	2.19
	R5	24.87	3.04	87	48.02
**MDA-MB-436**	A10+I+Ct	29.26	2.20	92	213.66
	A5	22.61	4.49	88	75.43
	D5	21.74	7.13	68	15.24
	R5	25.82	4.32	98	122.67
**SkBr3**	A10	21.41	3.67	90	36.13
	A5	18.94	3.84	89	44.35
	D5	21.80	4.61	93	67.31
	R5	20.27	6.63	85	23.04
