# Supplementary material for: An Effect of Culture Media on Epithelial Differentiation Markers in Breast Cancer Cell Lines MCF7, MDA-MB-436 and SkBr3
Source: Medicina (Kaunas). 2018 Mar 30;54(2):11. doi: 10.3390/medicina54020011 (PMC6037242; doi:10.3390/medicina54020011)
Supplement: Supplementary file 1 [file medicina-54-00011-s001.zip › Supplementary files_after final proofreading/Pirsko et al_SupplTable03_Gene info.pdf]

Pirsko V, Cakstina I, Priedite M, Dortane R, Feldmane L, Nakazawa-Miklasevica M, Daneberga Z, Gardovskis J, Miklasevics E. “An effect of culture media on epithelial differentiation markers in breast cancer cell lines MCF7, MDA-MB-436 and SkBr3”

Supplementary Table 3. Gene information

Category	Gene Name	Gene description	Reference number(s)
Mature luminal regulator (MLR)	AR	androgen receptor	NM_001011645, NM_000044
	ESR1	estrogen receptor 1	NM_000125, NM_001122740, NM_001122741, NM_001122742, NM_001291230, NM_001291241, NM_001328100, XM_011535543, XM_017010376, XM_017010377, XM_017010378, XM_017010379
	FOXA1	forkhead box A1	NM_004496
	GATA3	GATA binding protein 3	NM_001002295, NM_002051
	HES1	Hes family bHLH transcription factor 1	NM_005524
	PGR	Progesterone receptor	NM_000926, NM_001202474, NM_001271161, XM_006718858, XM_011542869
	TBX3	T-box 3	NM_005996, NM_016569
Luminal progenitor regulator (LPR)	ELF5	E74-like factor 5 (ets domain transcription factor)	NM_001243080, NM_001243081, NM_001422, NM_198381, XM_017017308, XM_017017309
	EZH2	Enhancer of zeste 2 polycomb repressive complex 2 subunit	NM_001203247, NM_001203248, NM_001203249, NM_004456, NM_152998, XM_017011817, XM_011515883, XM_005249962, XM_011515884, XM_011515885, XM_005249963, XM_011515886, XM_011515887
	FOXM1	Forkhead box M1	NM_001243088, NM_001243089, NM_021953, NM_202002, NM_202003, XM_011520932, XM_011520933,

			XM_011520935, XM_005253676, XM_011520930, XM_011520931, XM_011520934
	FOXQ1	Forkhead box Q1	NM_033260
	SOX9	SRY (sex determining region Y)-box 9	NM_000346
	VGLL1	Vestigial-like family member 1	NM_016267
Basal regulators (BR)	HEY1	hes-related family bHLH transcription factor with YRPW motif 1	NM_001040708, NM_001282851, NM_012258
	ID3	inhibitor of DNA binding 3, dominant negative helix-loop-helix protein	NM_002167
	IFI16	interferon, gamma-inducible protein 16	NM_001206567, NM_005531, XM_006711290, XM_005245127, XM_017001150, XM_017001149
	SNAI2	Snail family zinc finger 2	NM_003068
	TBX2	T-box 2	NM_005994, XM_011525159
	TP63	Tumor protein p63	NM_001114978, NM_001114979, NM_001114980, NM_001114981, NM_001114982, NM_003722, NM_001329148, NM_001329145, NM_001329146, NM_001329149, NM_001329150, NM_001329144, NM_001329964, XM_017007387, XM_005247844, XM_011513251
Luminal marker (LM)	EPCAM	Epithelial cell adhesion molecule	NM_002354
	ESR2	Estrogen receptor 2	NM_001437, NM_001040275, NM_001214902, NM_001271876,

			NM_001271877, NM_001291712, NM_001291723, XM_017021079, XM_017021080, XM_017021081, XM_017021082, XM_017021083, XM_017021084
	KRT18	Keratin 18	NM_000224, NM_199187
	KRT19	Keratin 19	NM_002276
	KRT7	Keratin 7	NM_005556, XM_017019294.1
	KRT8	Keratin 8	NM_001256282, NM_001256293, NM_002273
	MUC1	Mucin 1, cell surface associated	NM_001204294, NM_001204293, NM_001204292, NM_001204288, NM_001204287, NM_001204286, NM_001204285, NM_001018017, NM_001018016, NM_002456
	NAT1	N-acetyltransferase 1 (arylamine N-acetyltransferase)	NM_001160170, NM_001160171, NM_001160172, NM_001160173, NM_001160175, NM_000662, NM_001160176, XM_011544687, XM_011544688
	TMEM45B	Transmembrane protein 45B	NM_138788, NM_001331210, NM_001331211, NM_001331212, XM_017017188, XM_017017189
	SLC39A6	Solute carrier family 39 (zinc transporter), member 6	NM_012319, NM_001099406, XM_011525900, XM_011525901
	XBP1	X-box binding protein 1	NM_001079539, NM_005080
Basal marker (BM)	ERBB2	erb-b2 receptor tyrosine kinase 2	NM_001289938, NM_001289936, NM_001005862
	EGFR	Epidermal growth factor receptor	NM_005228
	FOXC1	Forkhead box C1	NM_001453
	ITGB3	Integrin, beta 3 (platelet	NM_000212

		glycoprotein IIIa, antigen CD61)	
	KRT16	Keratin 16	NM_005557, XM_170845
	KRT17	Keratin 17	NM_000422
	KRT6A	Keratin 6A	NM_005554
	KRTAP5-6	keratin associated protein 5-6	NM_001012416
	MIA	Melanoma inhibitory activity	NM_001202553, NM_006533
	PROCR	Protein C receptor, endothelial	NM_006404, XM_011528496.1
